# Evaluating the performance of propensity score matching based approaches in individual patient data meta-analysis

**DOI:** 10.1186/s12874-021-01452-1

**Published:** 2021-11-23

**Authors:** Fatema Tuj Johara, Andrea Benedetti, Robert Platt, Dick Menzies, Piret Viiklepp, Simon Schaaf, Edward Chan

**Affiliations:** 1 Department of Epidemiology, Biostatistics and Occupational Health, McGill University, Montreal, Canada; 2 Research Institute, McGill University Health Center, Montreal, Canada; 3 Centre for Clinical Epidemiology Sir Mortimer B. Davis, Jewish General Hospital, Montreal, Canada; 4 Department of Medical Registries, National Institute for Health Development, Tallinn, Estonia; 5 Department of Paediatrics and Child Health, Stellenbosch University and Tygerberg Children’s Hospital, Cape Town, South Africa; 6 Pulmonary Section, Rocky Mountain Regional Veterans Affairs Medical Center, Aurora, USA

**Keywords:** Observational studies, Bias, Confounding, IPD-MA, Propensity score matching

## Abstract

**Background:**

Individual-patient data meta-analysis (IPD-MA) is an increasingly popular approach because of its analytical benefits. IPD-MA of observational studies must overcome the problem of confounding, otherwise biased estimates of treatment effect may be obtained. One approach to reducing confounding bias could be the use of propensity score matching (PSM). IPD-MA can be considered as two-stage clustered data (patients within studies) and propensity score matching can be implemented within studies, across studies, and combining both.

**Methods:**

This article focuses on implementation of four PSM-based approaches for the analysis of data structure that exploit IPD-MA in two ways: (i) estimation of propensity score model using single-level or random-effects logistic regression; and (ii) matching of propensity scores (PS) across studies, within studies or preferential-within studies. We investigated the performance of these approaches through a simulation study, which considers an IPD-MA that examined the success of different treatments for multidrug-resistant tuberculosis (MDR-TB). The simulation parameters were varied according to three treatment prevalences (according to studies, 50% and 30%), three levels of heterogeneity between studies (low, moderate and high) and three levels of pooled odds ratio (1, 1.5, 3).

**Results:**

All approaches showed greater biases at the higher levels of heterogeneity regardless of the choices of treatment prevalences. However, matching of propensity scores using within-study and preferential-within study reported better performance compared to matching across studies when treatment prevalence varied across-studies. For fixed prevalences, a random-effect propensity score model to estimate propensity scores followed by matching of propensity scores across-studies achieved lower biases compared to other PSM-based approaches.

**Conclusions:**

Propensity score matching has wide application in health research while only limited literature is available on the implementation of PSM methods in IPD-MA, and until now methodological performance of PSM methods have not been examined. We believe, this work offers an intuition to the applied researcher for the choice of the PSM-based approaches.

## Background

When randomized control trials are not feasible, observational studies may be conducted to evaluate the association between a treatment of interest and relevant outcomes. Individual patient data from multiple observational studies can be combined and analyzed to obtain a pooled estimate of the treatment-outcome association [1]. Individual-patient data meta-analysis (IPD-MA) of observational studies allows enlarged sample sizes compared to individual studies, and may provide a more precise estimate of the treatment effect [2].

In IPD-MA of observational studies, treatment assignment is not random, which may cause an imbalance between the treatment and control groups in terms of important confounders or risk factors [3]. Traditional analytic strategies for the analysis of IPD-MA incorporate covariates into regression models to account for such imbalance between subjects, while also accounting for the correlation between subjects from the same study [4].

An alternative way to ensure a balance between treated and untreated groups is to use propensity scores. The propensity score is a probability of being treated conditional on the given observed covariates [5]. The Propensity score is a balancing score, which summarizes confounders in an efficient way.

Propensity scores can be used in a variety of ways including regression adjustment, weighting, stratification, and matching [6–8]. Matching on propensity scores has been shown to perform better in reducing bias compared to other propensity score techniques in individual studies [9].

Propensity score matching (PSM) has been used for the analysis of clustered data via several approaches: searching for a match from the entire pool of possible controls while ignoring the cluster level (“across clusters”), searching for a match only within the same cluster as the treated subject (“within clusters”), and finally, preferential within cluster matching, where first a suitable control is sought from within the same cluster, and if one is not found, then a control is sought from other clusters (“preferential within cluster”)[10, 11].

IPD-MA may be considered as clustered data, where the cluster is the study. However, there are important differences between IPD-MA and clustered data where these approaches have been previously evaluated. First, treatment prevalence may not vary greatly in clustered data [12]. However, IPD-MA may include studies from very different contexts and correspondingly, treatment prevalence may vary greatly, even for example from 0 to 100% - because some treatments are not available in some study centres. Second, heterogeneity may be greater in the IPD-MA context, since characteristics of the patient population, exposure and outcome definitions, and other study-level characteristics vary across studies [13].

In most meta-analyses, studies in which no subjects received the treatment of interest would be excluded, or alternatively network meta analysis may be considered. If study-level covariates are important, control subjects from the same study may be more similar to treatment subjects. Bias may arises because of the exclusion of treated subjects. Treated subjects might get excluded from the analysis if matched controls are not found. In that case, finding a control with a similar propensity score from a different study may be a reasonable choice. Moreover, given that some treatments may not have been available in some studies, a better match may be available by considering subjects in different studies. On the other hand, matching across studies may not ensure an appropriate match. This is because when study-specific confounders are strong, the estimated propensity scores may vary greatly across studies. More importantly - the same subject could have different PS in different settings/studies, so subjects from different studies with the same PS are not inherently comparable. A compromise is to use preferential within-study matching, which first searches for a suitable control from the same study and if none is found searches for a suitable control from a different study. In this work, we explore the consequences of various matching strategies.

Thus, the objective of this work was to systematically evaluate the performance of propensity score matching-based approaches to estimate the odds ratio that characterizes the treatment-outcome association in the context of IPD-MA via simulation study. Our work was based on an IPD-MA that investigated the treatment success of various drugs for multiple drug resistant tuberculosis (MDR-TB) [12].

This paper is structured as follows. Section 2 briefly describes the individual-patient dataset. In section 3, we describe a simulation study, which was used to investigate the performance of the PSM-based techniques. This section discusses the data generation procedure, simulation schemes, PSM-based approaches, and data analysis. In section 4, we present the results of the simulation study. Finally, in section 5, we summarize our findings.

## Methods

### Description of individual patient dataset

Our data generation relied on a real-life IPD-MA dataset that evaluated the associations between MDR-TB treatments and treatment success. Only patients with confirmed MDR-TB were included. Patients with extensively drug-resistant tuberculosis (XDR-TB) were excluded. We refer to this dataset as the MDR-TB-IPD. Ahuja et al. described in detail the study identification, selection and participation in IPD-MA [12].

The MDR-TB-IPD contained both individual-level and study-level information including patients’ age, sex, HIV status and acid fast bacilli (AFB) as well as smear status at the start of the MDR-TB treatment regimen, history of TB, cavitation status of the patient, information on 15 pharmacological agents, resistance status for each treatment, and the treatment outcome.

Specifically, the MDR-TB-IPD dataset is comprised of 31 studies with a total of 9290 patients. The number of patients per study varied from 25 to 2211, while the median number of patients across studies was 104 (see Table 1). The study-specific mean age of patients varied from 7 to 48 years, with a median mean age of 41 years across studies. Overall, most patients were males with the proportion of male ranging from 47% to 96% across studies. AFB smear status varied from 70% to 100%, while the median proportion of AFB smear positive across studies was 75%. Most patients were HIV-negative; indeed, 22 studies had no HIV-positive patients. Treatment prevalence for the drug “ethionamide” varied from 0 to 100% - because some treatments were not available in some study centres.

**Table 1:** Characteristics of study participants by study and overall

Study	N	Age mean (SD)	Male N. (*%*)	Smear N. (*%*)	HIV N. (*%*)	Ethionamide N. (*%*)
Ahujan	823	41.4 (12.0)	561 (68.2)	509 (70.8)	488 (74.4)	394 (47.9)
Avenda	72	36.3 (15.3)	43 (59.7)	67 (93.1)	1 (1.4)	13 (18.1)
Burgos	48	47.2 (14.8)	32 (66.7)	36 (75.0)	11 (22.9)	17 (35.4)
Chan	203	42.0 (14.4)	116 (57.1)	203 (100.0)	0 (0.0)	124 (61.1)
Chiang	125	46.1 (15.2)	90 (72.0)	109 (93.2)	0 (0.0)	57 (45.6)
Cox	77	36.9 (11.2)	47 (61.0)	76 (98.7)	0 (0.0)	72 (93.5)
Garcia	47	47.6 (16.4)	26 (55.3)	42 (89.4)	0 (0.0)	22 (46.8)
Granic	104	40.3 (19.5)	61 (58.7)	75 (74.3)	1 (100.0)	47 (45.2)
Koh	155	40.9 (14.4)	82 (52.9)	131 (84.5)	0 (0.0)	74 (47.7)
Lung	99	46.1 (16.2)	74 (74.7)	78 (80.4)	0 (0.0)	47 (47.5)
Migliori	101	39.4 (14.7)	61 (60.4)	80 (79.2)	6 (6.2)	46 (45.5)
Mitnic	732	31.1 (12.0)	436 (59.6)	508 (71.1)	8 (1.2)	495 (67.6)
Narita	81	40.2 (11.8)	55 (67.9)	0 (0.0)	41 (54.7)	26 (32.1)
Palmer	114	35.3 (13.7)	54 (47.4)	108 (94.7)	0 (0.0)	8 (7)
Pasvol	45	36.0 (16.7)	21 (50.0)	29 (74.4)	0 (0.0)	25 (55.6)
Pena	25	41.2 (13.3)	24 (96.0)	25 (100.0)	0 (0.0)	0 (0)
Perez	34	42.1 (12.4)	21 (61.8)	34 (100.0)	0 (0.0)	0 (0)
Quy	157	39.5 (11.4)	121 (77.1)	157 (100.0)	4 (2.5)	0 (0)
Riekstina	1027	42.3 (12.7)	780 (75.9)	269 (68.1)	32 (3.9)	0 (0)
Robert	45	41.7 (15.6)	24 (53.3)	33 (73.3)	9 (25.7)	18 (40.0)
Schaaf	39	7.0 (5.4)	20 (51.3)	9(33.3)	6 (20.7)	30 (76.9)
Seung	1427	43.9 (15.4)	117 (82.4)	142 (100.0)	0 (0.0)	0 (0)
Shim	1364	42.8 (14.9)	1014 (74.3)	927 (68.0)	1 (0.1)	0 (0)
Shin	608	35.8 (11.3)	506 (83.2)	497 (85.8)	5 (0.8)	450 (74.0)
Shirai	61	46.4 (11.9)	46 (75.4)	0 (0.0)	0 (0.0)	40 (65.6)
Tabars	43	44.4 (19.1)	27 (62.8)	42 (97.7)	0 (0.0)	0 (0)
Tupasi	170	39.2 (12.4)	106 (62.4)	107 (67.7)	0 (0.0)	40 (23.5)
Vander	43	32.9 (18.3)	32 (74.4)	0(0.0)	0 (0.0)	1 (2.3)
Vander	2211	36.6 (10.8)	1383 (62.6)	1390 (69.7)	571 (38.4)	2211 (100)
Viiklepp	284	43.0 (13.6)	201 (70.8)	153 (53.9)	9 (3.4)	0 (0)
Yimkim	211	39.3 (15.8)	124 (58.8)	0 (0.0)	0 (0.0)	0 (0)
Median	104	41	63	75	0	41
*P*_25_	48	37	59	68	0	0
*P*_75_	207	43	74	94	5	51

N.(%): The number and percentage of patients in each study.

Median, *P*_25_ and *P*_75_ indicates the median, 25th and 75th Percentiles which have been calculated for the number of patients, mean age of patients, proportion of male patients, proportion of smear status, HIV status across studies, and proportion of treatment Ethionamide

We conducted a simulation study to evaluate the performance of propensity score matching-based approaches to estimate treatment effects in the context of MDR-TB-IPD.

### Simulation study

**Fig. 1:**
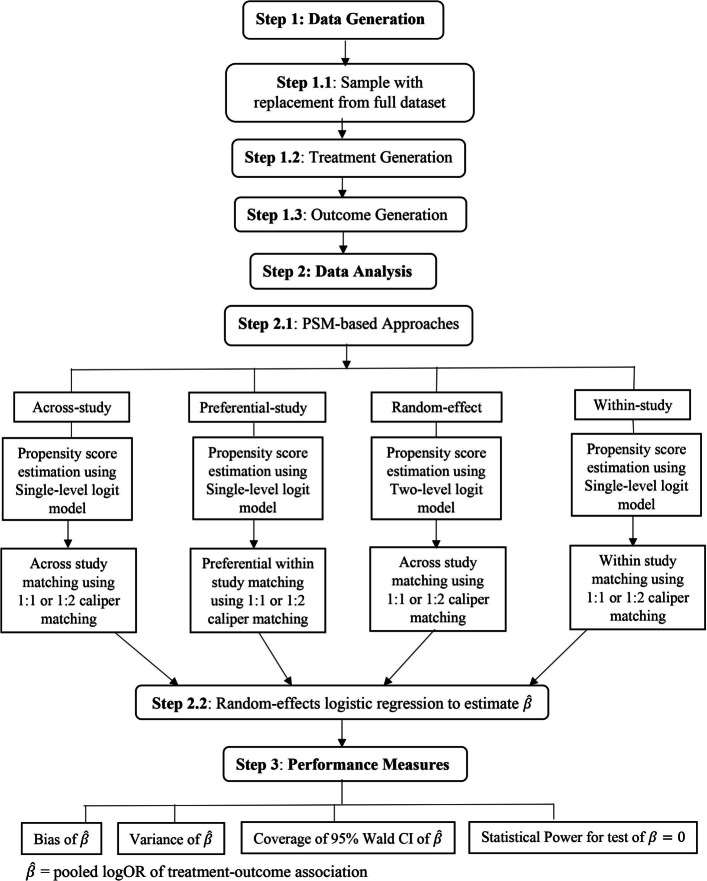
Step-by-step simulation study

Figure 1 gives an overview of the steps of the simulation study, which we describe generally here, and in detail below. We generated our two-level data structure where *N* individual-level units, indexed by *i* (*i*=1,2,⋯,*n*_*j*_), are nested in J second-level units (studies), indexed by *j* (*j*=1,2,3,⋯,*J*). Our confounder variables, *X*_*ij*_ = (age, sex, HIV status, and smear status) were generated by sampling with replacement from the MDR-TB-IPD dataset. Next, we generated our binary treatment variable, *Z*_*ij*_, which depended on the confounder values. Finally, we generated a binary outcome variable, *Y*_*ij*_, depending on the covariates and treatment, and a study-specific random intercept and slope.

For each generated data set, we implemented several propensity score matching strategies that differed in how the propensity score was estimated and in how the matching was accomplished (details below). At the end of this step, we had a data set that was ready for analysis. We estimated the pooled odds ratio for the association between our binary treatment and treatment success (our binary outcome) via a random effects logistic regression with random intercept and slope by study.

We repeated these steps 1000 times for each scenario (where we varied the data generation parameters), and then considered the following metrics of performance: the bias and variance of the pooled log odds ratio as well as coverage of the 95% confidence interval around this parameter, and the power to detect a statistically significant effect.

### Data generation details

#### Step 1.1: confounder generation

We designed our simulation study to mimic the observed data in different scenarios. That is, instead of generating covariates as realizations from random variables, we used the same distribution of covariates as observed in the MDR-TB-IPD dataset. From this data set, we selected 4 potential confounders and 1 treatment. We considered 4 individual-level covariates that regression analyses indicated had a significant association with both the treatment and outcome variables [14]: age, sex, smear and HIV status. We chose ethionamide as a representative treatment because treatment prevalence of this drug varied from 0 to 100%, depending on the study. Moreover, we kept the same two-level structure (patients nested in studies) as observed in the MDR-TB-IPD. Thus, to generate the data sets, we first selected a sample of 5000 subjects, sampled with replacement from the original MDR-TB-IPD data (*n*=9290), ignoring the study level. Then, studies with fewer than 25 subjects were excluded from the analysis. This is because each study MDR-TB-IPD has at least 25 subjects.

#### Steps 1.2 and 1.3: treatment and outcome generation

Next, we generated the treatment and outcome variables, using those selected covariates, and including random-effects (details below). To inform our treatment variable generation, we fitted a generalized linear mixed model (GLMM) with ethionamide as the outcome, the four covariates (age, sex, smear and HIV status) as independent variables and a random intercept for study, using the MDR-TB-IPD (See Table 2). Similarly, we fitted a GLMM with treatment success as the outcome, the four covariates and treatment with ethionamide as independent variables, and a random intercept and random slope for treatment, to inform our outcome generation (See Table 2).

**Table 2:** Parameter estimates and standard deviation of the random intercepts and slopes obtained by fitting generalized linear mixed models to the MDR-TB-IPD

	Treatment Model	Outcome Model
Covariate	$\log {\boldsymbol {\hat {\alpha }}} (SE)$	$\log {\boldsymbol {\hat {\gamma }}} (SE)$
Age	-0.01 (0.00)	-0.03 (0.01)
Sex	0.03 (0.07)	0.04 (0.16)
AFB smear status	-0.42 (0.08)	-0.47 (0.17)
HIV status	-0.80 (0.01)	-0.83 (0.22)
Ethionamide	–	0.30 (0.33)
SD of random intercept	0.7	0.9
SD of random slope for treatment	–	0.04
(ethionamide)		

#### Step 1.2 treatment generation

We generated a single binary treatment variable chosen to mimic ethionamide (see Table 1), that depended on the individual-level covariates. We considered three treatment generation mechanisms:

#### Treatment prevalence according to studies

We generated the treatment variable by considering the actual treatment prevalence of ethionamide in each study in the MDR-TB IPD dataset, which varied from 0 to 100% (See Table 1). To generate the treatment variable, we estimated 31 study-specific intercepts separately according to the treatment prevalence of ethionamide in each study.

Thus, the binary treatment variable, was generated from a single-level logit model:

1 $$ logit(s_{{ij}})=\alpha_{0j}+ \mathbf{X_{{ij}}} \boldsymbol\alpha,  $$

where *s*_*ij*_=*P*(*Z*_*ij*_=1|*X*_*ij*_) is the probability of receiving treatment conditional on the individual-level covariates (*X*_*ij*_),***α*** is the vector of fixed effects corresponding to these covariates, and *α*_0*j*_ is the study-level fixed effect.

#### 50% treatment prevalence

In the MDR-TB-IPD dataset, overall nearly 50% of patients took the treatment ethionamide. Thus, we considered fixing the average treatment prevalence at 50%, and estimating single intercept, *α*_0_, by considering the overall treatment proportion, *P*(*Z*_*ij*_=1|*X*_*ij*_,*U*_*j*_)=0.50 while generating the treatment variable. Therefore, a binary treatment variable was generated from a two-level random effects logit model:

2 $$ logit(s_{{ij}})=\alpha_{0} + \mathbf{X_{{ij}}} \boldsymbol\alpha + u_{0j},  $$

where *s*_*ij*_=*P*(*Z*_*ij*_=1|*X*_*ij*_,*U*_*j*_) is the probability of receiving treatment conditional on the individual-level covariates and study-level random-effect, $u_{0j} \sim N(0,\sigma _{0j}^{2})$.

#### 30% treatment prevalence

We were interested in how a lower treatment prevalence that might increase the number of potential controls would impact our results. Thus, in this scenario, we estimated a single intercept, *α*_0_, by considering the overall treatment proportion, *P*(*Z*_*ij*_=1|*X*_*ij*_,*U*_*j*_)=0.30, all other details as in the 50% case.

For each treatment generation scenario, the study level fixed effects were the parameter estimates obtained from the generalized linear mixed treatment model estimated using the MDR-TB-IPD data, described in subsection Steps 1.2 and 1.3, as was the variance of the random intercept for the second and third scenario. Therefore, we set the standard deviation of the random effect *σ*_0*j*_=0.7 and the coefficients: log(*α*_1_)=−0.01, log(*α*_2_)=0.03, log(*α*_3_)=−0.42, and log(*α*_4_)=−0.80 for each of treatment generation mechanism, and estimated the probability of receiving ethionamide. Treatment status was generated by using a Bernoulli distribution with the estimated treatment probability.

#### Step 1.3 outcome generation

We generated the outcome variable from a GLMM such that the probability of treatment success was conditional on the individual-level characteristics (*X*_*ij*_), treatment *Z*_*ij*_, and study-level random effects:

3 $$ \begin{aligned} logit[P(Y_{{ij}}=1|\mathbf{X_{{ij}}},U_{j})] = \gamma_{0} + u_{0j} + (\beta + u_{1j}) Z_{{ij}} + \mathbf{X_{{ij}}} \boldsymbol{\gamma}, \end{aligned}  $$

where *P*(*Y*_*ij*_=1|*X*_*ij*_,*U*_*j*_) is the probability of treatment success; $u_{0j} \sim N(0,\sigma _{0j}^{2})$ are study-specific random intercepts; *β* is the logarithm of the true pooled odds ratio for the treatment effect; $u_{1j} \sim N(0,\sigma _{1j}^{2})$ are the study-specific random slope for treatment; *Z*_*ij*_ is the binary treatment variable (generation described earlier); *X*_*ij*_ is the matrix of individual-level covariates; and, ***γ*** is the vector of fixed-effect coefficients.

We used the parameter estimates and standard deviations of the random intercept and slope, from a generalized linear mixed outcome model fitted to the MDR-TB-IPD data (see subsection Step 1.2 and 1.3) to generate the outcome variable. Thus, we set the coefficients: log(*γ*_1_)=−0.03, log(*γ*_2_)=0.04, log(*γ*_3_)=−0.47, and log(*γ*_4_)=−0.83, and the standard deviations of the random-intercept *σ*_0*j*_=0.7, and the random-slope, *σ*_1*j*_=0.04. To mimic the MDR-TB-IPD dataset where treatment success was [*Y*_*ij*_=1]=90*%*, we generated the probability of treatment success considering, *P*(*Y*_*ij*_=1|*X*_*ij*_,*U*_*j*_)=0.9, in the logit model. Finally, we converted the estimated probability to a binary outcome variable using a Bernoulli distribution.

#### Varying simulation

In further scenarios, we generated data that incorporated mild, moderate and high degrees of heterogeneity across studies, and with a null, moderate and strong effect of treatment. Therefore, in our simulation, for each treatment generation mechanism and outcome generation considered we considered three SDs of the random intercepts, *σ*_0*j*_={0.7,1.4,2.1} and random slopes, *σ*_1*j*_={0.04,0.08,0.12}, and three pooled odds ratio, *β*={1,1.5,3} [15], for a total of 3×3×3 = 27 distinct scenarios were investigated (see Table 3).

**Table 3:** Varying features in our simulation study

Features	
Treatment prevalence	According to study
	50%
	30%
SD of the Random intercept	0.7
for outcome generation	1.4
	2.1
SD of the random slope	0.04
for outcome generation	0.08
	0.12
Pooled odds ratio for the	1
treatment effect, when	1.5
generating the outcome	3

### Data analysis

#### Step 2.1: pSM-based approaches in the context of iPD-MA

To estimate the treatment effect, we considered several propensity score matching approaches, previously applied in clustered data contexts.

In the first step, a logistic regression model was specified to estimate the propensity score. As IPD-MA data exhibits a two level-data structure, we considered both a single-level and two-level logit model. In the second step, suitable control subjects with similar propensity scores were sought for each treatment subject. We considered three matching criteria to find an appropriate control subject for each treated subject within a two-level data structure: (i) across study matching; (ii) within study matching; and (iii) preferential within study matching. Thus, we considered four PSM-based approaches for the analysis of IPD-MA:

- Single-level logit model to estimate PS followed by matching of PS across studies (across-study)
- Single-level logit model to estimate PS followed by matching of PS within studies (within-study)
- Single-level logit model to estimate PS followed by preferential within study matching of PS (preferential-study)
- Two-level random-effect logit model to estimate PS followed by matching of PS across studies (random-effect)

For each PSM based approach, we evaluated one-to-one (1:1) caliper matching with a caliper=0.2 [16]. We describe each approach in detail next.

#### Single-level logit model to estimate pS followed by matching of pS across studies (across-study)

This approach considers a single-level logistic model to estimate the propensity score, which ignores the clustering of subjects within studies [17]:

4 $$ logit(s_{i})= \alpha_{0}+ \mathbf{X_{{ij}}} \boldsymbol{\alpha},  $$

where *s*_*i*_=*P*(*Z*_*ij*_=1|*X*_*ij*_) is the conditional probability of receiving treatment, *X*_*ij*_ is the matrix of individual-level covariates and *α* is the vector of parameters corresponding to the covariates.

After the propensity scores were estimated, matching across studies with replacement was considered [18]. We used 1:1 caliper matching that identified pairs (or triplets) of treatment and control subjects whose difference between propensity scores was not more than 0.2 times the standard deviation of the logit of the propensity scores.

#### Single-level logit model to estimate pS followed by matching of pS within studies (within-study)

In this approach, the single-level logit model 4 was used to estimate the propensity score, which ignored the multi-level structure of the dataset[19, 20]. For each treatment subject, control subjects from the same study were sought. When a suitable control subject was not found for a treatment subject, the unmatched treatment subject was dropped from the analysis.

#### Single-level logit model to estimate pS followed by preferential within study matching of pS (preferential-study)

For this strategy, the propensity scores were estimated from a single-level logit model 4 [21]. First, we searched for a control subject within the same study, but if an appropriate control subject was not found from the same study, then a control was sought in other studies.

#### Two-level random-effect logit model to estimate pS followed by matching of pS across studies (random-effect)

In this approach, we used a generalized linear mixed effects model for estimating the propensity scores [22]:

5 $$ logit(s_{{ij}})= \alpha_{0}+ \mathbf{X_{{ij}}} \boldsymbol{\alpha}+ u_{0j},  $$

where *s*_*ij*_=*P*(*Z*_*ij*_=1|*X*_*ij*_,*U*_*j*_) is the conditional probability of receiving treatment; *X*_*ij*_ is the matrix of individual-level covariates. It is the vector of parameters corresponding to individual-level covariates; $u_{0j} \sim N(0,\sigma _{0j}^{2})$ are the study-level random-effects in the logit model. After the estimation of propensity scores, 1:1 caliper matching algorithm was implemented across the studies.

#### Step 2.2: estimating the treatment effect via random-effects logistic regression model

Once the matched dataset was formed, a random-effects logit model was estimated:

6 $$ logit[P(Y_{{ij}}=1|\mathbf{X_{{ij}}}, U_{j})]= \beta_{0}+u_{0j}+(\theta+u_{1j}) Z_{{ij}}+\omega+ \hat{s}_{{ij}},  $$

where *Z*_*ij*_ denotes the treatment status of *i*^*th*^ subject within *j*^*th*^ study, *exp*(*θ*) is the pooled odds ratio to be estimated, $u_{0j} \sim N(0,\sigma _{0j}^{2})$ is the study-specific random intercept, $u_{1j} \sim N(0,\sigma _{1j}^{2})$ is the study-specific random slope, $\hat {s}_{{ij}}$ is the estimated propensity score from PSM model and *ω* is the corresponding parameter of the estimated propensity score.

### Performance measures

For each simulated data set (*n*=1000), we obtained the treatment effect estimate and its standard error, 95% Wald confidence interval, and *p*-value. From this output, we computed the mean bias, the variance of treatment effect estimates, coverage of 95% Wald confidence interval, and statistical power. In the next section, we present the data summary by descriptive statistics of MDR-TB-IPD dataset and results of the simulation study.

## Results

Table 4 presents a comparison of the performance of four PSM-based strategies in terms of mean bias (bias) of the log pooled odds ratios (OR: 3 and 1.5) under three treatment generation mechanisms, and for varying treatment effects, and study-level heterogeneity (SD of the random intercept: 0.7, 1.4, and 2.1 and SD of the random slope: 0.04 and 0.08). Please note that: simulation results for odds ratio = 1 and SD of random slope = 0.12 are reported in the separate file “Supplemental Tables.pdf”.

**Table 4:** Simulation results: Mean bias of log(OR) by PSM-based approaches and data generation features

			Treatment Prevalence According to Study				50% Treatment Prevalence				30% Treatment Prevalence			
OR	SD ^*c*^	SD ^*b*^	Across	Preferential	Random	Within	Across	Preferential	Random	Within	Across	Preferential	Random	Within
	(*σ*_1*j*_)	(*σ*_0*j*_)	Study	Study	Effect	Study	Study	Study	Effect	Study	Study	Study	Effect	Study
3	0.04	0.7	0.09	0.00	-0.17	-0.03	0.11	0.09	0.01	0.05	0.15	0.06	0.04	0.06
		1.4	0.23	0.06	0.06	0.03	0.32	0.25	0.10	0.15	0.45	0.22	0.13	0.20
		2.1	0.46	0.22	0.25	0.17	0.58	0.38	0.17	0.19	0.76	0.23	0.18	0.18
	0.08	0.7	0.09	-0.01	-0.17	-0.03	0.10	0.08	0.00	0.04	0.14	0.05	0.03	0.05
		1.4	0.22	0.05	0.05	0.02	0.30	0.24	0.09	0.14	0.44	0.21	0.13	0.19
		2.1	0.45	0.21	0.24	0.16	0.56	0.37	0.15	0.17	0.74	0.22	0.17	0.16
1.5	0.04	0.7	0.07	-0.03	-0.21	-0.07	0.10	0.04	0.00	0.04	0.15	0.06	0.04	0.06
		1.4	0.23	0.03	0.00	-0.02	0.31	0.29	0.09	0.12	0.43	0.13	0.13	0.18
		2.1	0.43	0.14	0.11	0.05	0.58	0.45	0.16	0.18	0.75	0.19	0.18	0.14
	0.08	0.7	0.07	-0.04	-0.22	-0.07	0.10	0.10	-0.01	0.03	0.14	0.05	0.03	0.05
		1.4	0.22	0.02	-0.01	-0.02	0.29	0.28	0.07	0.10	0.42	0.19	0.12	0.17
		2.1	0.42	0.13	0.09	0.03	0.56	0.44	0.14	0.16	0.73	0.17	0.17	0.13

OR indicates pooled odds ratio used for simulation

^c^SD is the standard deviation, *σ*_1*j*_ of study specific random slope, *u*_1*j*_

^b^SD is the standard deviation, *σ*_0*j*_, of study specific random intercept, *u*_0*j*_

Across-study indicates consideration of Single-level logit model with across-study matching

Prefer-study indicates consideration Single-level logit model with preferential-within study matching

Random-effect indicates consideration of Random-effect logit model with across-study matching

Within-study indicates consideration of Single-level logit model with within-study matching

**Fig. 2:**
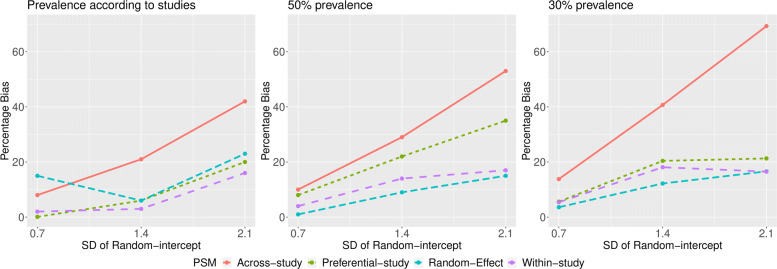
Percent Mean Bias of the estimated log(OR) by PSM-based approach and heterogeneity in treatment prevalence (left: prevalence varied from 0 to 100%, according to that observed in each study; middle: 50% prevalence; right: 30% prevalence) when the true pooled OR =3

No matter how treatment prevalence was generated, the across-study approach estimated the most biased treatment effect, while the random-effect and within-study approaches estimated the least biased treatment effects (see Fig. 2). The preferential-study approach performed similarly to the within-study and random-effect approaches when treatment prevalence was generated according to studies, and when it was set at 30% (see Fig. 2). When treatment prevalence was set at 50%, the preferential-study approach estimated treatment effects that were more biased than those estimated via the within-study and random-effect approaches, but not as biased as those estimated via the across-study technique (see Fig. 2). For each approach, bias increased as the variance of the random intercept increased. Results did not vary appreciably with magnitude of the pooled OR, or standard deviation of the random slope.

**Fig. 3:**
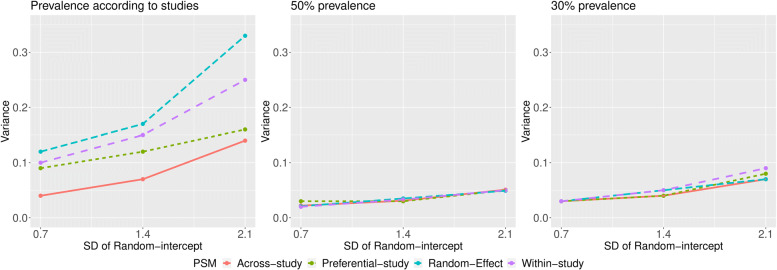
Variance of estimated log(OR) by PSM-based approach and heterogeneity in treatment prevalence (left: prevalence varied from 0 to 100%, according to that observed in each study; middle: 50% prevalence; right: 30% prevalence) when the true pooled OR =3

PSM-based techniques were also compared in terms of variance of log(OR) estimates (Table 5). For the consideration of treatment prevalence according to study, the variances of the estimates produced by the across-study approach were lower than those produced by other approaches, no matter the magnitude of the true pooled OR, or the variances of the random intercepts or random slopes. Variances increased as the variance of the random intercepts increased for all approaches, but the variance produced by the random-effect approach increased more drastically (see Fig. 3).

**Table 5:** Simulation results: variance of log(OR) by PSM-based approaches and data generation features

			Treatment Prevalence According to Study				50% Treatment Prevalence				30% Treatment Prevalence			
OR	SD ^*c*^	SD ^*b*^	Across	Preferential	Random	Within	Across	Preferential	Random	Within	Across	Preferential	Random	Within
	(*σ*_1*j*_)	(*σ*_0*j*_)	Study	Study	Effect	Study	Study	Study	Effect	Study	Study	Study	Effect	Study
3	0.04	0.7	0.04	0.09	0.12	0.10	0.02	0.03	0.02	0.02	0.03	0.03	0.03	0.03
		1.4	0.07	0.12	0.17	0.15	0.03	0.03	0.04	0.03	0.04	0.04	0.05	0.05
		2.1	0.14	0.16	0.33	0.25	0.05	0.05	0.05	0.05	0.07	0.08	0.07	0.09
	0.08	0.7	0.05	0.07	0.13	0.11	0.02	0.03	0.02	0.02	0.03	0.03	0.03	0.03
		1.4	0.07	0.12	0.18	0.14	0.04	0.03	0.04	0.03	0.05	0.04	0.05	0.05
		2.1	0.14	0.17	0.34	0.25	0.05	0.05	0.05	0.05	0.06	0.08	0.06	0.08
1.5	0.04	0.7	0.04	0.07	0.12	0.12	0.02	0.03	0.03	0.02	0.03	0.03	0.03	0.03
		1.4	0.07	0.12	0.17	0.16	0.04	0.06	0.05	0.06	0.04	0.05	0.04	0.06
		2.1	0.12	0.20	0.33	0.32	0.05	0.06	0.05	0.06	0.07	0.08	0.07	0.09
	0.08	0.7	0.05	0.08	0.12	0.12	0.02	0.03	0.03	0.02	0.02	0.03	0.03	0.03
		1.4	0.06	0.13	0.16	0.16	0.05	0.06	0.05	0.06	0.04	0.05	0.04	0.06
		2.1	0.13	0.19	0.32	0.32	0.06	0.06	0.05	0.06	0.06	0.08	0.07	0.09

OR indicates pooled odds ratio used for simulation

^c^SD is the standard deviation, *σ*_1*j*_ of study specific random slope, *u*_1*j*_

^b^SD is the standard deviation, *σ*_0*j*_, of study specific random intercept, *u*_0*j*_

Across-study indicates consideration of Single-level logit model with across-study matching

Prefer-study indicates consideration Single-level logit model with preferential-within study matching

Random-effect indicates consideration of Random-effect logit model with across-study matching

Within-study indicates consideration of Single-level logit model with within-study matching

When treatment prevalence’s were assumed to be fixed to 50% and 30%, the approaches produced estimates with comparable variability for low variance of the random intercepts (Fig. 3). As the variance of the random intercepts increased, the variance produced by each PSM-based approach increased slightly and maintained similar extent of variability.

Coverage of the 95% Wald confidence intervals estimated when each PSM approach was used to estimate the pooled OR. When treatment prevalence was according to study, coverage ranged from 82% to 96% (see Table 6). The across-study approach had coverage closest to nominal levels while the within-study approach had the lowest coverage. There was a slight drop in coverage for the across-study approach, while the other approaches showed a slight increase in coverage when heterogeneity increased. When treatment prevalence was set at 50% and 30% and variances of the random intercepts was low, coverage was relatively similar across PSM approaches. However, coverage decreased markedly for the across-study approach as the variance of the random intercepts increased. Preferential-study, random-effect and within-study all showed near nominal but increasing to over coverage as the variance of the random intercepts increased.

**Table 6:** Simulation results: Coverage of 95% confidence interval to estimate log(OR) by PSM-based approaches and data generation features

			Treatment Prevalence According to Study				50% Treatment Prevalence				30% Treatment Prevalence			
OR	SD ^*c*^	SD ^*b*^	Across	Preferential	Random	Within	Across	Preferential	Random	Within	Across	Preferential	Random	Within
	(*σ*_1*j*_)	(*σ*_0*j*_)	Study	Study	Effect	Study	Study	Study	Effect	Study	Study	Study	Effect	Study
3	0.04	0.7	93	90	92	88	86	92	96	92	88	95	98	95
		1.4	92	93	93	91	77	91	97	92	66	89	96	91
		2.1	87	93	93	91	64	95	98	98	50	95	97	97
	0.08	0.7	93	90	92	88	87	92	96	93	89	96	97	95
		1.4	92	93	93	91	79	91	98	93	67	89	96	91
		2.1	87	93	94	92	68	96	99	98	53	95	98	98
1.5	0.04	0.7	93	89	90	89	86	92	95	92	86	93	96	92
		1.4	92	94	93	91	78	92	96	93	68	95	96	92
		2.1	88	94	94	94	66	96	99	98	51	97	97	98
	0.08	0.7	93	89	90	89	87	92	95	93	86	93	96	94
		1.4	92	94	93	92	80	94	97	94	70	92	96	93
		2.1	89	94	94	93	69	97	99	99	55	97	98	98

OR indicates pooled odds ratio used for simulation

^c^SD is the standard deviation, *σ*_1*j*_ of study specific random slope, *u*_1*j*_

^b^SD is the standard deviation, *σ*_0*j*_, of study specific random intercept, *u*_0*j*_

Across-study indicates consideration of Single-level logit model with across-study matching

Prefer-study indicates consideration Single-level logit model with preferential-within study matching

Random-effect indicates consideration of Random-effect logit model with across-study matching

Within-study indicates consideration of Single-level logit model with within-study matching

Table 7 shows the statistical power and type 1 error of estimated log(OR) for varying treatment prevalence according to studies. Power to detect a statistically significant effect was highest for the across-study approach, and lowest for the random-effects approach. For the consideration of 50% and 30% treatment prevalence’s, type 1 error was very inflated for the across-study approach and increased as the variance of the random intercepts increased. For the other approaches, type 1 error was between 1-11%. Power to detect a statistically significant effect decreased as the variance of the random intercepts increased. Within-study and preferential-study approaches had better power than the random-effect approach.

**Table 7:** Simulation results: Statistical Power and type I error of log(OR) by PSM-based approaches and data generation features

			Treatment Prevalence According to Study				50% Treatment Prevalence				30% Treatment Prevalence			
OR	SD ^*c*^	SD ^*b*^	Across	Preferential	Random	Within	Across	Preferential	Random	Within	Across	Preferential	Random	Within
	(*σ*_1*j*_)	(*σ*_0*j*_)	Study	Study	Effect	Study	Study	Study	Effect	Study	Study	Study	Effect	Study
3	0.04	0.7	100	95	83	90	100	100	100	100	100	100	100	100
		1.4	100	89	79	84	100	99	100	99	100	99	99	99
		2.1	99	83	72	73	100	97	98	95	100	92	94	91
	0.08	0.7	100	95	83	90	100	100	100	100	100	100	100	100
		1.4	100	88	79	84	100	99	100	99	100	100	99	99
		2.1	98	83	72	72	100	96	98	94	100	91	94	90
1.5	0.04	0.7	64	50	22	47	92	80	69	80	90	77	72	76
		1.4	60	38	29	37	87	60	49	56	90	49	48	57
		2.1	53	27	22	25	88	32	29	24	91	30	28	25
	0.08	0.7	63	49	21	46	92	79	68	77	89	77	70	75
		1.4	59	36	27	36	86	58	47	53	89	60	46	55
		2.1	52	27	21	24	87	29	26	22	90	28	26	23

OR indicates pooled odds ratio used for simulation

^c^SD is the standard deviation, *σ*_1*j*_ of study specific random slope, *u*_1*j*_

^b^SD is the standard deviation, *σ*_0*j*_, of study specific random intercept, *u*_0*j*_

Across-study indicates consideration of Single-level logit model with across-study matching

Prefer-study indicates consideration Single-level logit model with preferential-within study matching

Random-effect indicates consideration of Random-effect logit model with across-study matching

Within-study indicates consideration of Single-level logit model with within-study matching

**Fig. 4:**
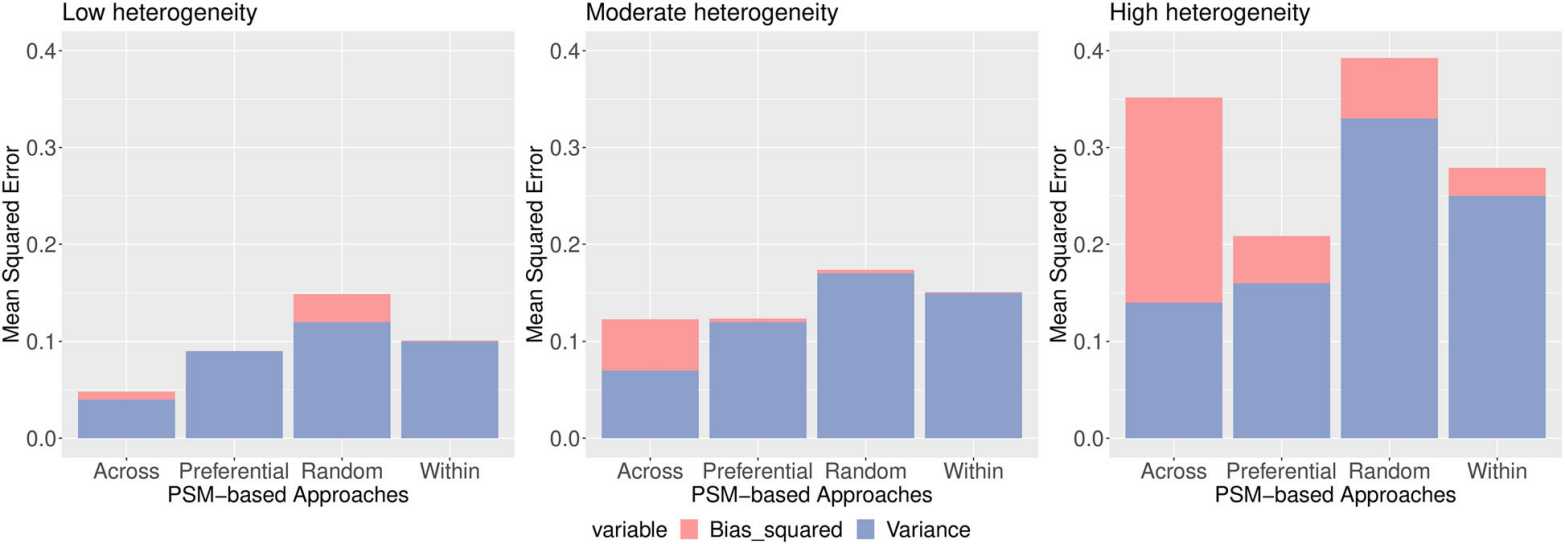
MSE of estimated log(OR) by PSM-based approach and heterogeneity (left: low; middle: moderate; right: high) when prevalence varied from 0 to 100%, according to that observed in each study and the true pooled OR =3

PSM-based approaches were also compared on the basis of mean squared errors (MSEs) in the context of low, moderate and high heterogeneous situation. For the treatment prevalence according to studies for the pooled odds ratio 3, we can see that the across-study approach produced the lowest MSE when the data exhibited low heterogeneity (see Fig. 4). Across-study, within-study and preferential study had lower MSEs compared to random-effect approach for moderate heterogeneous situation. For high heterogeneity, both the across-study and preferential-study obtained lowest MSEs.

**Fig. 5:**
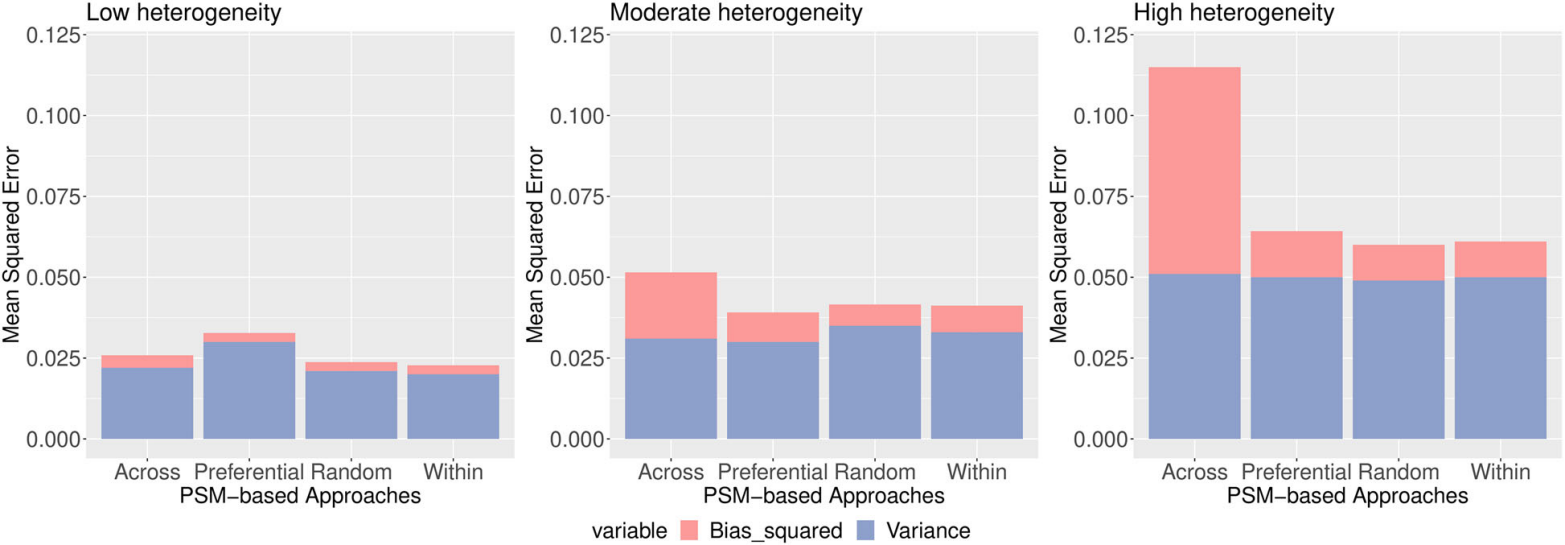
MSE of estimated log(OR) by PSM-based approach and heterogeneity (left: low; middle: moderate; right: high) when 50% treatment prevalence and the true pooled OR =3

For 50% treatment prevalence, within-study and random-effect approaches produced lower MSEs in the low, moderate and high heterogeneity contexts (see Fig. 5).

**Fig. 6:**
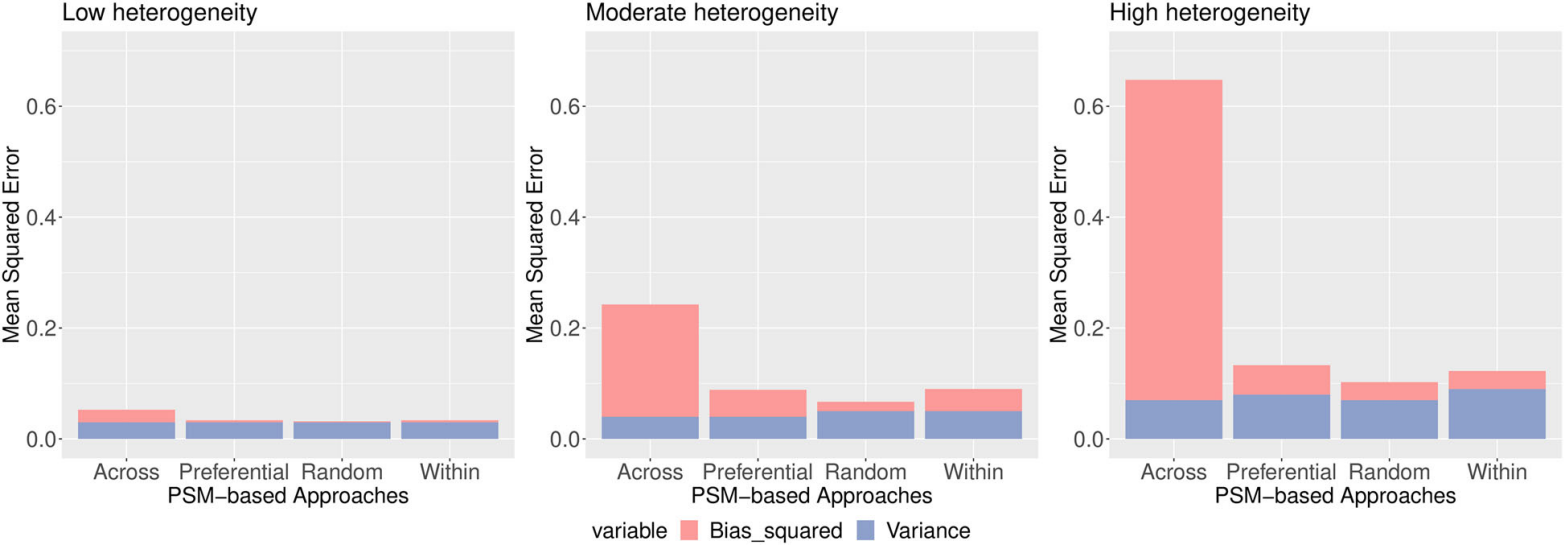
MSE of estimated log(OR) by PSM-based approach and heterogeneity (left: low; middle: moderate; right: high) when 30% treatment prevalence and the true pooled OR =3

For 30% treatment prevalence, within-study, random-effect and within-study approaches produced lower MSEs in the low, moderate and high heterogeneity contexts (see Fig. 6).

## Discussion

Individual-patient data meta-analysis is considered as the ’gold standard’ of meta-analyses because of the potential to reduce heterogeneity by standardizing many aspects of the data analysis and offering numerous analytical benefits. IPD meta-analysis of observational studies must carefully attempt to resolve the problem of confounding. Using propensity scores to reduce the potential bias caused by an imbalance in important covariates may be a promising approach in this context. We considered several propensity score matching based approaches for the analysis of IPD-MA of observational studies. We investigated the performance of these approaches through a simulation study, based upon the real-life MDR-TB-IPD dataset [12].

When treatment prevalence varied according to studies (from 0 to 100%), and data exhibited low heterogeneity among studies, the single-level logit model to estimate the PS with across study matching (across-study approach) produced an estimated pooled log odds ratio with lower bias and variance. This approach also reported reasonable coverage and statistical power. Despite of having higher type I error, across-study approach may still be recommended as it produced lower bias, variance, MSE, and attained reasonable statistical coverage when data exhibit low heterogeneity.

However, when the IPD-MA exhibited moderate heterogeneity between studies, both the single-level logit model to estimate the PS with across study matching (across-study technique) and single-level logit model to estimate the PS with preferential-within study matching (preferential-matching approach) reported lowest MSEs, reasonable coverage and power compared to single-level logit model to estimate the PS with within-study matching (within-study approach) and two-level random-effects logit model to estimate the PS with across study matching (random-effect approach). In particular, across-study matching produced lower variance whereas preferential-study reported much lower bias. In contrast, in the presence of higher heterogeneity among studies, the single-level logit model to estimate the PS with preferential-within study matching (preferential-study approach) produced lowest MSE with lower bias and variance.

When there was little variation of treatment prevalence across studies and overall prevalence was 50%, single-level logit model to estimate the PS with preferential-within study matching (preferential-study approach) showed poor performance compared to other three approaches, when heterogeneity was low. In contrast, the single-level logit model to estimate the PS with across study matching (across-study approach) reported weak performance (showed higher MSEs) compared to other three PSM-based techniques when heterogeneity was moderate or high. Likewise, the single-level logit model to estimate the PS with across study matching (across-study approach) showed very poor performance compared to other three PSM-based approaches for any extent of heterogeneity when a lower treatment prevalence (for example, 30%) was considered.

Our results aligned in some situations with the results presented for clustered data structures [21]. In our simulations, when treatment prevalence varied from 0 to 100%, preferential-study matching performed better than other PSM-based techniques when between study heterogeneity was high. Similar performance of the preferential-study technique has been observed for two-stage clustered data in the presence of very small and large clusters [21]. In two-stage clustered data, bias increased significantly due to lots of unmatched subjects mainly when clusters were relatively small and within cluster matching was used [23]. Likewise, in our study, the within-study approach reported higher bias when treatment prevalence was varied according to studies. This is because some studies had a treatment prevalence of 0, or a very low prevalence. With such a data structure, implementation of within-study matching may result in many unmatched treatment subjects, which produce greater biases. When important cluster-level variables are ignored, a single level logit model to estimate the PS with across-cluster matching makes it challenging to obtain unbiased estimates of the treatment effect. Likewise, in our study performance of the across-study approach reported greater biases at high heterogeneous situation (see Figs. 4, 5, 6). Arpino and Cannas [21] reported less bias when a random-effects logistic model was used to estimate the propensity scores, which is similar to the pattern we observed when treatment prevalence was fixed at 50% and 30%. Fox et al. [24] used propensity score matching techniques on the MDR-TB-IPD data that we used in our simulation. They compared several analytic strategies empirically and found that propensity score matching based techniques achieved adequate covariate balance between treated and untreated individuals. They also reported that matching within studies and matching across studies achieved the closest covariate balance compared to covariate based multivariate techniques.

While we investigated a wide range of scenarios, these were not exhaustive, and the results seen here may not apply to scenarios not investigated. Moreover, we did not compare the PSM based approaches to traditional analytic approaches as this is beyond the scope of paper. Additionally, we made several simplifying decisions, for example, we considered only a subset of confounders. Other clinically important covariates may influence treatment allocation or outcome in the MDR-TB-IPD dataset [25]. We also assumed that propensity score models were correctly specified. Incorrect specification of the propensity score model may produce a biased estimate of the treatment effect of interest [26].

The within study approach may not be suitable if most of the treatment subjects get excluded because of poor control match in some studies. In such situations, across-study matching or preferential within study or random effect approach may be adopted. Across study approach considers IPD-MA as a single dataset and provides greater opportunity to find a suitable control subject for each treatment subjects. There are other propensity score matching approach in literature, for example nearest neighbor matching, that selects a control subject for each treatment subject even if the propensity scores differ a lot between treatment and control subjects [27]. This approach may end up with treatment and control subjects that are very different, which could induce biases [27].

We considered only a single treatment, whereas there may be interest in evaluating the effects of several treatments. Propensity scores for many treatments are possible, though how they would perform in this context is unknown. However, Brown et al. (2020) proposed an approach for applying propensity score matching when multi treatments are under consideration [28]. In this study, only binary outcomes were considered. However, further investigations are needed to extend these results to continuous and time-to-event outcomes. Finally, We have arbitrarily set heterogeneity to levels we describe as “low”, “moderate”, and “high”.

It may not be reasonable to expect that all observational studies included in an IPD-MA measure the same list of potential confounders, in the same way (e.g. on the same scale). In such situations, two analytical strategies may be considered: (i) study-specific propensity score models could be considered; or (ii) only the common set confounders across studies could be considered, which may lead to unmeasured confounding [29]. Further study is required to investigate the performance of both strategies.

## Conclusions

This work allows us make some recommendations regarding what kind of PSM-based technique is expected to perform the best depending on treatment prevalence and interstudy heterogeneity for analyzing IPD-MA of observational studies. If treatment prevalence for the drug of interest varies greatly, and the IPD-MA exhibits lower heterogeneity between studies, then using a single-level logit model to estimate the PS with across study matching can be used to form matched treatment-control pairs. However, for higher heterogeneity between studies, using a single-level logit model to estimate the PS with preferential within study matching should be used to form the matched dataset. On the other hand, if the treatment prevalence for the drug of interest has less variation and a preferential-study or within-study matching or using a random-effects approach can be considered to perform analysis of IPD-MA no matter the extent of heterogeneity between studies. The extent of heterogeneity can be defined through standard meta-analysis. Higgins (2002) suggested one useful statistic: I-squared for quantifying inconsistency [13]. This describes the percentage of the variability in effect estimates that is due to heterogeneity rather than sampling error (chance). PSM-based approaches could be decided based on their bias, variance and MSE.

Despite the comprehensive application of propensity score matching in health research, only limited literature is available on the implementation of PSM methods in IPD-MA, and until now methodological performance of PSM methods have not been examined. We believe, this work offers an intuition to the applied researcher for the choice of the PSM-based approaches based on a variety of treatment prevalence scenario and different heterogeneity of IPD meta-analysis of observational studies.

## Data Availability

The datasets used and/or analysed during the current study are available from the corresponding author on reasonable request.

## Abbreviations

PSM: Propensity score matching
IPD-MA: Individual-patient data meta-analysis
MDR-TB: Multiple drug resistant tuberculosis
XDR-TB: Extensively drug-resistant tuberculosis
AFB: Acid fast bacilli
HIV: Human immunodeficiency virus
GLMM: Generalized linear mixed model
PS: Propensity score
MSE: Mean squared errors
OR: Odds ratio
